# QUEST FOR EXCELLENCE VII Washington, DC February 6–8, 1995

**DOI:** 10.6028/jres.100.022

**Published:** 1995

**Authors:** Cap Frank, Robert E. Chapman

**Affiliations:** Office of Quality Programs, National Institute of Standards and Technology, Gaithersburg, MD 20899-0001

## 1. Introduction

To form a foundation for a national quality improvement campaign, American industry and government jointly established and support the Malcolm Baldrige National Quality Award. Created by public law in 1987, the Award promotes an understanding of quality excellence, greater awareness of quality as a crucial competitive element, and the sharing of information and successful strategies through conferences such as the annual Quest for Excellence and a variety of publications. There are three categories in which companies may compete: manufacturing, service, and small business.

The Baldrige Award Criteria (continuously evolving through each of the 7 years of the program’s existence) provide a framework against which companies assess their quality programs. Business leaders note that in addition to being the basis for Award selection and for giving feedback to award applicants, the Malcolm Baldrige National Quality Award Criteria hold a significant leadership role in strengthening U.S. competitiveness by:

- helping to raise quality performance practices and expectations;
- facilitating communication and sharing among and within organizations of all types based upon a common understanding of key quality and operational performance requirements; and
- serving as a working tool for planning, training, and assessment.

The National Institute of Standards and Technology’s Office of Quality Programs, which manages the Malcolm Baldrige National Quality Award program, describes the goal of the Award Criteria as assisting American industry to deliver ever-improving value to customers, resulting in improved marketplace performance and improved overall company operational performance.

The Criteria have as their foundation a collection of core values and concepts. Excerpting from the 1994 Malcolm Baldrige National Quality Award Criteria publication, some of these are: customer-driven quality, effective senior leadership, continuous improvement, employee participation and development, long-range outlook, management by fact, partnership development, corporate responsibility and citizenship. These core values and concepts are embodied in the seven categories of the Award Criteria: Leadership, Information and Analysis, Strategic Quality Planning, Human Resource Development and Management, Management of Process Quality, Quality and Operational Results, and Customer Focus and Satisfaction.

As a component of its outreach and education mission and to ensure that the Baldrige Award continues to be a catalyst for change, the NIST Office of Quality Programs is available to assist states and localities to adopt Baldrige-based criteria and application assessment procedures for their own recognition programs. As of the date of the conference, 42 state and local quality award programs were in operation in 30 states. Most were modeled after the Baldrige Award. To further broaden the impact of quality practices, the Baldrige program is experimenting with expanding quality criteria to education and health care with full-scale pilot projects underway this year in both areas.

Each year the Quest for Excellence Conference provides a forum for quality conscious business leaders worldwide to hear and question the most recent Baldrige Award recipients. They come to this conference to learn about the quality journeys of Award-winning companies and how to adapt and implement total quality management practices to their own organizations. Conference participants view the current and prior Award winners as quality practices benchmarks for American industry.

During the 3 day conference, representatives from each of the three Award recipients—AT&T Consumer Communications Services (headquarters: Basking Ridge, New Jersey) and GTE Directories Corporation (headquarters: Dallas/Ft. Worth, Texas), winners in the Service Category, and Wainwright Industries (headquarters: St. Peters, Missouri) a Small Business winner—addressed full conference plenary sessions and smaller group discussion workshops on each of the criteria categories. All sessions set aside significant time for audience questions.

The most successful Quest for Excellence Conference ever held, based on written participant feedback, concluded with two important new panels. Senior leaders from the 1994 Baldrige-winning companies presented lessons learned from experiences associated with applying for and winning the Baldrige Award during one panel. The other panel addressed “Quality Pays” and included a keynote address and presentations by senior representatives from past Baldrige Award recipients.

In his keynote address commencing this year’s conference, Deputy Secretary of Commerce David J. Barram, a former vice president of Worldwide Corporate Affairs and Public Policies for Apple Computer, observed, “Whether you are an industrial supplier or a small supplier, whether you provide a service or manufacture a product, I can think of no better tool to help you outpace the competition than the Baldrige National Quality Award Criteria. According to quality pioneer Joseph Juran, ‘Total quality management consists of those actions needed to get to world class quality.’ Right now the most complete list of those actions is contained in the criteria for the Baldrige Award.”

Mr. Barram emphasized, “The sharing of information by winning companies is truly remarkable. They take seriously their charge to be advocates of quality. They willingly share the secrets of their success in quality management. Because they believe, as I do, that this knowledge will make our economy and our nation stronger and that’s good for everyone’s business. To date the winners have given more than 15,000 presentations reaching thousands of organizations, an awesome and powerful force for change and improvement. And something I believe has made a big difference in the quality and culture in American business.”

Reflecting on Deputy Secretary Barram’s keynote address, GTE Directories president Earl Goode added, “The Baldrige Award was used to inspire a shared goal and the criteria were used to take a snapshot of a point in time and an ongoing measure of progress over time.” Similar sentiment has been voiced by most previous Award winners and many other companies that have used the Criteria as their prime internal quality improvement road map.

This Conference Report highlights the “best practices” presentations by senior representatives of the three winning companies. The report is organized, as was this year’s Quest for Excellence Conference, by the Baldrige Criteria categories beginning with the Leadership Category (because of their strong interrelationship, the conference organizers merged the categories for process management and business results into one session: management of process quality/quality and operational results).

## 2. Leadership

The Baldrige Award Criteria describe leadership as senior executives’ personal leadership and involvement in creating and sustaining a customer focus, clear values and expectations, and a leadership system that promotes performance excellence. Leadership is also how the values and expectations are integrated into the company’s management system, including how the company addresses its public responsibilities and corporate citizenship. Dr. Curt W. Reimann, the individual most responsible for the Baldrige Criteria and director of NIST’s Office of Quality Programs, noted that one of the morals taken from the experiences of the 22 Baldrige Award winners is that, “Thunderous leadership from the corner office may be necessary, but unless it’s turned into a system of some kind and an ongoing organization, it perhaps does not achieve the kinds of ends that are important.”

Top executives from this year’s winners all note that an effective leader has to know how and when to let go. AT&T Consumer Communications Services president Joseph P. Nacchio explained, “I don’t tell my people what to do, rather I only tell my people what not to do. I tell them not to lose customers, don’t violate our values, and don’t break any laws. The rest of it is their business. The power of our processes is more important than any single personality.”

Mr. Nacchio echoed Earl Goode, GTE Directories president, and Arthur D. (Don) Wainwright, Wainwright Industries CEO, when he observed that a leader, “Starts with a vision and communicates it well, makes sure the vision is based on a set of values that represents the leader’s beliefs, positions the customers prominently in the vision triage and then builds business processes that support the vision. It isn’t enough to know that our customers and our associates are the key to success, and it isn’t enough to know that strong business results reflect high customer satisfaction and high associate satisfaction, and it isn’t enough to know that the integration of quality processes and the business processes produce business excellence. The leadership team must translate that knowledge into plans and programs. We must model the kinds of value-based behavior described in our common bond to the way we do our jobs.”

All three companies described leadership as trust, vision, communication, and support. They all saw leadership challenges as: continue to lead from the front lines rather than from headquarters; to communicate, face-to-face, person-to-person; and to trust the judgment of empowered employees and the proven quality processes.

Earl Goode added, “Consistency, over time, of message, of focus and direction is one of the central parts of being a leader.” According to Mr. Goode, key GTE Directories’ leadership actions are:

1. Put a stake in the ground.
  Putting a stake in the ground is creating a clear vision “We will win the Baldrige Award in 1994 [announced in January 1991], and set a compelling goal—such as 100 % customer satisfaction through quality. Communicate and explain the priorities to all in the corporate family—for instance, by using interactive television broadcasts to 50 sites for on the spot Q & A and information dissemination.”
2. Get out of the way.
  “Empower people through teams, provide effective governance structure—hire the best, train them in their jobs, turn them loose to get the job done.”
3. Have the courage to stay the course.
  “Do what you’ll say you’ll do, listen, communicate, listen, communicate; don’t give up!” (check on progress towards the goal, and redirect if necessary). “When you do what you say you will do, then employees will know that your commitment is real. Have the courage to let go and let your people determine how best to get the job done. It’s up to leadership to insure that the organization is moving together towards the same destination, and it’s up to the people to figure out the best way to get there.”

Don Wainwright told the audience he believed in leadership through emotion, “Leadership needs an emotional commitment to a vision and to the people that make the vision happen. If leadership is sensitive to the needs of others and expresses it in appropriate terms, it’s very contagious. People are much more capable than they think they are, and are actually willing to do more than you think they will.”

In a phrase, the leadership approach of Wainwright Industries, AT&T Consumer Communication Services and GTE Directories is: Vision, Focus, Communication.

## 3. Information and Analysis

The Baldrige Criteria for information and analysis provide a yardstick for measuring the effectiveness of the use and management of data and information to support customer-driven performance excellence and marketplace success.

According to GTE Directories’ senior vice president, Douglas C. LaVelle, information determines how the company serves its stakeholders (stakeholders being customers, GTE shareholders, employees, suppliers, and the general public). A customer satisfaction measurement program is used to determine what is important to GTE Directories customers, how well the company is performing, and how the company can do better. GTE Directories collects data on its seven drivers of customer satisfaction—product quality, documentation, ad content, sales, business relationships, customer service, and value. By analyzing the gaps in internal organization performance and benchmarking against industry standards, the direction of the company can be reset.

Information collection for Susan K. Cutler, CPA, Wainwright Industries controller, is guided by five indicators, each with 1–3 year strategic goals: safety, internal customer satisfaction, external customer satisfaction, six sigma quality, and business performance. The Mission Control room at Wainwright Industries is truly information central, or as CEO Don Wainwright described it, “a cross between a carnival tent and an auditor’s retreat.” Trends for quality and performance, and for each customer’s monthly satisfaction index scores are posted along with trends for key quality measures, stretch targets for exceeding customer expectations, and weekly feedback reports. Mission Control provides a concrete visual link with the five indicators by posting, for all to see, including customers and competitors, recordable accidents, the associate suggestion rate, internal customer satisfaction, external customer satisfaction, internal (defective) parts per million, sales, and net income. Information is also collected for Wainwright operational indicators such as equipment utilization, overtime, value added waste stream, reactionary maintenance, inventory turns, and delivery performance. Business support indicators such as financial data (for example: accounts payable and receivable), requisition cycle time, turnover, and invoice accuracy are also posted in their most current form. Data are collected for competitive comparison and benchmarking purposes by examining trends of key indicators, tracking progress along strategic goals, and networking to collect benchmark data. According to Ms. Cutler, “Wainwright Industries is fanatical about collecting information on best practices, from customers and outstanding competitors.”

Beth Bronner, AT&T vice president of domestic consumer communications services, noted, “Most correct decisions are locked upon the power of information. It is a matter of determining which is the right information, the irrefutable data which everyone in the organization has confidence in.” As such, according to AT&T CCS chief information and technical officer Alan Jones, AT&T CCS gathers and analyzes information based on the three value added drivers—customer, people, and economic factors. Customer Value Added (CVA), People Value Added (PVA), and Economic Value Added (EVA) data are collected every day.

Data from the CVA index reflect how AT&T CCS customers perceive the overall value of CCS products and services compared to those of the competition. CVA considers all of the customer satisfiers: call quality, customer service, billing, reputation, and price. PVA measures each associate’s (employee’s) perception of leadership, job satisfaction, and diversity issues as components of stated objectives and associate expectations. Scientific surveys of all associates annually and random samples quarterly are used to collect PVA data. The EVA index measures how well the individual product lines meet the projected return. It also measures financial data such as return on investment and profit. AT&T CCS defines EVA as the return on capital minus the cost of that capital (i.e., shareowner surplus after everything is paid off, including the cost of capital).

Information management (especially data gathering and analysis) is the foundation of the AT&T CCS competitive advantage. Ms. Bronner of AT&T CCS observed, “Massive amounts and endless varieties of data are gathered to improve the AT&T value position. The trick is to collect the right data. The worst case is to have piles of information and reports that no one knows what to do with.”

AT&T CCS uses the analyses to generate key learnings, stretch goals, and Direct Measures of Quality (DMOQ) figures. Key learnings are the company’s building blocks for performance improvement, while stretch goals for unit costs, market share, cycle times and billing accuracy raise organizational and unit performance levels. Direct Measures of Quality track progress along stretch goals. Amplifying the sentiments of her session co-presenters, AT&T’s Beth Bronner closed the session with, “Do the right thing, at the right time, and use the right tools.”

## 4. Strategic Quality Planning

Steve Gibbons, Association for Quality and Participation president, the session’s moderator, quoted the Baldrige Criteria by noting that, “Strategic quality planning is how the company sets its strategic directions, how it determines key plan requirements, and how the plan requirements are translated into an effective performance management system.”

Michael Simms, Wainwright Industries plant manager noted that the Wainwright Industries definition of planning 10 years ago was “Yes sir, Mr. Customer, you want it tomorrow, you got it.” This direct unencumbered approach worked well for a number of years when customer requirements and products were relatively few in number. As the product line expanded, the company began to self destruct (i.e., fall apart under the tremendous weight of unchecked expansion). Mr. Simms observed, “It is only recently, with the help of the Baldrige Criteria and insights gained from previous Baldrige winners, especially after attending past Quest for Excellence Conferences, that Wainwright Industries reached what it called its planning milestone, understanding that the plan for the future has to align what’s important to the customer with what’s important to the company.” As a result four alignment factors were developed: best price, consistent profitability (all that can be expected realistically is a fair return over the long term), product quality, and on-time delivery.

Coupled with the four alignment factors were five key indicator categories derived from a company-wide employee survey. In descending ranking order they are: safety, internal customer satisfaction, external customer satisfaction, six sigma quality (defined as 3.4 defective parts per million parts, or six standard deviations from the norm for defective parts), and business performance.

At Wainwright Industries business performance planning projects growth and plant capacities, capital expenditures, and net income while targeting markets and customers. As Mr. Simms mentioned, “The Wainwright strategy is that by doing a good job in the first four categories, strong business performance will automatically occur.”

AT&T CCS’s strategic goals are to reduce time to market and insure that products and services satisfy customers “right out of the gate.” Effective planning is needed to meet these strategic goals. Beth Bronner explained the AT&T CCS keys to strategy planning as: leadership must align the organization around the customer; processes must support customer needs; a consistent framework is needed for measuring success and failure; decision-making is encouraged; and the organization must be continuously reinvented with a focus on the customer.

Ms. Bronner continued, “Leadership is a key ingredient to strategic quality planning at AT&T CCS and must make its presence felt every day by being a participant, decision maker, visionary, communicator, and motivator. By clearly and effectively communicating goals and objectives, leadership insures that everyone receives the same strategic messages at the same time.”

AT&T CCS leaders believe that plans to reinvent the company must be a major component of strategic planning. But as Ms. Bronner noted, “Reinvention should not emanate from crisis or major events, but rather it must be viewed as a way of life, continuously reinventing one’s organizational processes [in a systematic, rational manner through strategic quality planning]. AT&T CCS is constantly listening to its customers and aligning all of the processes with the changing needs of the consuming public. As change is a very real way of life, any company which is unwilling or unable to reinvent itself for the short, middle, and long terms is destined to fall behind and allow its own inertia to penalize its customers, its people, and its bottom line.”

According to George Lieb, vice president—strategic planning and business development of GTE Directories, “The core of GTE’s strategic quality planning is to learn ‘What do our customers want.’ GTE Directories has learned over the last four years that its customers want value, strong business relationships, and outstanding customer service. The data were used to produce four business priorities, forming the basis for the strategic quality planning process—provide and demonstrate value to the customer, build business relationships, enhance customer service, improve cost effectiveness to enhance competitiveness (to ensure a return on investment for shareowners). Every corporate goal, strategy, and action plan must contribute to one or more of these priorities.”

Until a few years ago, GTE Directories regarded the strategic plan as confidential, to be shared with only a few key executives. Mr. Lieb noted, “Today, strategic planning is seen as everyone’s job. Now we realize all employees must understand the strategic objectives. We cannot expect anyone to make progress toward our goals if we don’t provide the road map that tells where we’re going. So now the strategic plan details are communicated to all levels of the corporation.”

The old way of strategic planning for GTE Directories was to have parallel planning tracks for the business plan and a quality plan. Mr. Lieb observed, “Three years ago a new mentality was discovered that integrated quality planning throughout the business planning process by bringing cross functional work teams and regional management councils earlier into the planning process.”

GTE Directories formulates long-term goals and a strategy to achieve those goals, based on bottom up employee internal input and external customer needs, supplier capabilities, and a variety of economic and technological risk factors. The company also takes a long-range view of the key competitive issues impacting their industry.

After action plan deployment, GTE Directories uses what it calls a “balanced scorecard approach” to measure action plan performance. The bottom line is not the only measure, rather one of six key measures: customer satisfaction, advertiser base growth, financial performance, employee satisfaction, recycled content, and supplier quality.

## 5. Human Resource Development and Management

Steve Gibbons, Association for Quality and Participation president noted that the Baldrige category for human resource development and management sets a yardstick for measuring how the workforce is enabled to develop and utilize its full potential and aligned with the company’s performance objectives. “This category also provides criteria to determine how companies build and maintain an environment conducive to performance excellence, full participation, and personal/organizational growth.”

According to AT&T CCS president Joseph Nacchio, in 1993/94 his company spent more than $100 million training associates, about a 20 % increase in funding compared to the previous 2 years. “AT&T CCS has done this while facing the same pressures as other telecommunication companies. Pressures that resulted in industry wide announcements of more than 100,000 layoffs in just the past 24 months. But we are not facing those pressures in the same ways as other companies. Our alliance for employee growth and development provides training of new skills before the old skills are obsolete. The workplace of the future brings our union and nonunion associates together to reshape the business, not just to negotiate contracts.”

To Anne Fritz, AT&T CCS vice president of human resources, “innovation is essential to human resources. Too often executives perceive innovation as something that is product specific, like re-engineering a manufacturing process. In fact the greatest innovation must take place with human management systems.”

The change in corporate culture from a hierarchical structure with a command and control foundation and a focus on internal objectives to process teams, customer focus, diversity, and coaching was prompted by the 1984 divestiture, globalization, and dramatically rapid technological and economic changes. The old system of planned change and a homogeneous workforce held no advantages in a new global economy which rewarded organizational leanness, diversity, and innovation. AT&T found itself in need of introspection and reinvention into a more entrepreneurial mold. Its workforce had to become equally flexible and entrepreneurial, and the corporate human resource development and management approach also had to be reassessed and, ultimately, retooled.

According to Ms. Fritz, AT&T CCS performed this cultural change by creating what it calls “Our Common Bond” program. The common bond was a corporate-wide dedication to helping customers. The common bond program goals were to breed respect for individuals and build the highest standards of integrity throughout the company. The vehicle for change was encouraging teamwork rather than individual performance. The corporate goal was innovation, both for its own sake and for the sake of moving the company forward. The slogan became “continually reinventing ourselves.” The foundation for the change was an increase in the role of education and training. AT&T CCS met the challenge by increasing its investment in education and training by 58 % between 1991 and 1994.

Rewards and recognition for individual and team results and behavior are another element of associate empowerment and satisfaction. AT&T CCS has 36 formal programs and multiple ad hoc spot recognition programs. In conjunction with its reward/recognition program, AT&T CCS attempts to transform the traditional work systems to also foster associate satisfaction. AT&T CCS uses innovation to create a model of the workplace of the future and addresses the “here and now” with associate-focused benefits such as flexible work design, work/family solutions, and career transition resources.

Ms. Fritz summarized the AT&T CCS approach to human resources development and management as ingraining in the corporate culture beliefs such as:

- innovation is as vital to human resources management as to any business discipline,
- human performance systems must lead change, not respond to it,
- reward, recognition, and compensation systems must be linked to specific, quantifiable customer results,
- associate empowerment, morale, and motivation can increase even in times of downsizing.

For GTE Directories to achieve its goal of 100 % customer satisfaction, it recognizes that its employees must be motivated, informed, involved, well trained, and satisfied. As company president Earl Goode has noted on a number of occasions, “our employees are not simply our most important asset, they are the company.” To GTE Directories vice president of human resources R. Bryant Byrd, the core of his company’s human resource management and development program is employee involvement in quality improvement teams and self-managed work teams. He noted, “Continuous quality improvement is a key human resources objective. We are always seeking feedback on our human resources programs and processes. We measure their effectiveness, and through external benchmarking, we compare ourselves with other companies and reevaluate our strategies as required.”

The quality improvement teams are a significant aspect of GTE Directories’ overall quality program. The teams encourage and foster many important quality improvement ideas from the mainstream, everyday operations. Since 1988, 100 employees have been trained as processes management facilitators to assist with the running of the quality improvement teams.

The 17 self-managed work teams create a climate of empowerment and optimism, while at the same time offering the organization a much more flexible and open work environment. Mr. Byrd noted, “The teams are responsible for hiring, work scheduling, performance management, job training, budget control, and other day-to-day business functions.”

Employee feedback is collected through employee satisfaction and opinion surveys. GTE Directories looks at the survey results to identify trends and priority actions. Focus groups are conducted to learn more about the key issues raised in the surveys and establish quality improvement teams to address any quality related issues. Results are also benchmarked with a consortium of major United States companies and against other available benchmark data. Mr. Byrd noted, “Based on the benchmarking results and our own internal assessments, we refine our employee survey and the process begins again.” GTE Directories currently is moving from a 2 year cycle to an annual survey. A change Mr. Byrd noted, “Which will help the company identify emerging issues and adapt to changes in the workforce more quickly.” The results of the employee and internal customer surveys are folded into an action plan which will be reflected in the human resource management process and in employee training and recognition programs.

According to Mr. Byrd, “A wide range of employee performance recognition and rewards are used to motivate our employees to support our quality improvement efforts and to encourage superior performance and are in two broad areas ranging in style from quality improvement spot awards to annual awards.” The two broad areas for the awards/recognition program are quality awards and performance recognition. Mr. Byrd emphasized, “Some of these recognition activities are informal and immediate and support the team-building culture. Spot recognition examples as the ‘Caught in the Act’ and ‘Quality Co-worker’ programs encourage employees to recognize their peers for simple everyday activities that demonstrate teamwork or good customer service.” Other reward and recognition programs are more formal and scheduled, for example, appreciation dinners, a sales incentive trip (this year to Sydney, Australia), the President’s Production Trophy, and the President’s National Quality Team Award. All employees are recognized for participation in quality improvement teams, and one employee is honored annually as the quality advocate of the year for quality improvement and leadership. All rewards and recognition support quality performance, excellence, and individual dedication in a team environment. All winners are recognized in the GTE Directories monthly publication, *Directions*, and in periodic employee bulletins.

At GTE Directories, Mr. Byrd observed, “We believe it’s important to maintain a working environment that’s conducive to the well being and professional growth of our people. We are proud of our work environment, we are proud of our employees, and they are proud to be a part of GTE Directories.”

Wainwright Industries CEO, Don Wainwright emphasized, “We are committed to creating an environment in which associates enjoy their work and consider their association with the company the best opportunity available to them in the world.” To carry out this vision, Dave Robbins, Wainwright Industries vice president, explained that empowerment is the key and that empowerment is the development of self-confidence, allowing for the acceptance of responsibility in the Wainwright workplace. The spirit of empowerment is the Wainwright Industries “mantra”, with management believing that empowerment builds self-confidence which encourages an acceptance of responsibility greater than the norm. Empowerment is the thread of the Wainwright culture and as such is woven throughout each associate’s work life cycle from new associate (employee) training through to cross training opportunities.

Wainwright Industries believes an informed associate is an empowered associate and training is a major component of the information exchange process. Cross-functional work teams and a training program which is 7 % of payroll serve to produce and maintain an empowered and motivated workforce. (Wainwright’s 7 % of payroll training budget compares favorably with three recent Baldrige winners which ranged from 3 % to 4.5 %.) Company president Nelson Wainwright noted, “Training is an investment, not an expense. Training is a superior investment. While equipment depreciates, an associate with training appreciates.”

Wainwright Industries starts the empowerment program with the new associate (employee) orientation process by involving senior leadership right from the start. CEO Don Wainwright spends an hour with each new employee within the first 2 weeks, to talk about vision, mission, values, the culture of the organization, job security with Wainwright Industries, and what it means to be a member of Team Wainwright.

In addition to new associate orientation, the Wainwright Industries training program covers topics such as problem solving, team performance interpersonal skills (basic values, human interaction), change and innovation, and organizational issues (primarily team building). Operator certification and statistical process control classes are also a part of the training mix. Cross training was broadened to foster promotional opportunities throughout the company.

The Wainwright Industries continuous improvement process (CIP) is a large part of worker empowerment. The CIP embodies the philosophy that overall organizational improvement is based on “improving many little things.” By involving everyone in the CIP and by streamlining the implementation approval process, “many little things” are discovered each week and changes are immediately adopted.

Management control was seen as a roadblock to CIP creativity, so the CIP approval process was replaced with “leadership that listens” and instantaneous responsiveness. The Wainwright commitment is that senior management will respond to any suggestion from any person within 24 hours and develop a plan of action to implement, or if possible, implement the suggestion within 72 hours. The Continuous Improvement Process has averaged at least one implementation of an associate suggestion per week since mid-1992 and is currently averaging 54.7 implemented suggestions (CIP’s) per associate annually. This compares with a benchmark of 15 implemented suggestions per employee per year, the typical Japanese company’s 12 and the typical American company’s 0.3.

By combining individual and team goals, for instance making safety the number one company priority (right next to customer satisfaction), dramatic results have been achieved. Video reenactment (which was a CIP implemented suggestion) is used for any recordable accident or potentially serious incident and the actors are the actual associates involved in the incident. They explain the accident, give the root cause analysis, and give their irreversible corrective action plan. The video is shown during breaks, at lunch, team meetings, and before and after work to stimulate safety thinking throughout the company and to insure that the accident will never happen again. As a result, both accident incidence and lost time have dropped dramatically since 1991, from a level just above the national average, to a 1994 level of one half the national average for incidents and one quarter for lost time.

Other results from the Wainwright Industries approach to human resources development and management are equally impressive. Since salary replaced other forms of compensation, attendance has been at 99 % or better for 10 consecutive years (workers get paid whether they come to work or not). At Wainwright Industries, satisfied employees produce satisfied customers—satisfied customers have increased from 50 % in 1992 to 98 % for 1994.

## 6. Management of Process Quality/Quality and Operational Results

Dr. Harry S. Hertz, deputy director of the NIST Office of Quality Programs and moderator for the panels discussing the two criteria categories covered in this section noted that, “Category five deals with all key processes of the organization, both product- and service-oriented processes as well as support and supplier processes, and that results must be reported relative those key processes. Categories five and six also show the very direct and important linkage between approach, deployment, and results.” Dr. Hertz also observed, “… that these two categories have undergone significant rewriting in our continuous improvement efforts in going from 1994 to 1995.” Category five in the 1995 Baldrige Criteria is titled Process Management and category six is now titled Business Results.

Pete Heiden, GTE Directories area vice president of publishing, noted, “… the conventional wisdom is that any new product or service begins with an idea. The reality is the process begins with information, the kind of information discussed during the Information and Analysis session earlier in this conference. Every month on an average, each sales representative consults with 200 individual customers. This broad and dense data base drives the front-end of new product development, new item development, or geographic rescoping.”

For GTE Directories, process management is one of its most widely used methodologies for quality improvement. Process management methodology is integrated to core business processes, support processes, and supplier processes. According to Mr. Heiden, “GTE Directories expects nothing less from our suppliers than what our customers expect from us.” Process management at GTE involves six discrete steps.

1. Map the particular process under review as it now “IS.”
2. Identify weaknesses, disconnects.
3. Conduct benchmarking.
4. Design the process as it “SHOULD” be.
5. Establish measures.
6. Implement.

Core business processes have shown dramatic improvement through process management methodology.

- Turnaround time has decreased from 40 days to 10.8 days over the last 2.5 years.
- Published errors have decreased from 450 published errors per million listings to under 375 published errors per million listings over the last 4 years.
- Bill processing productivity has eclipsed benchmarks by four to one each of the last 3 years.
- Customer complaints have decreased 46 % since 1992.
- The cost of customer billing adjustments has decreased 54 % since 1992.
- Major supplier ratings have steadily improved since 1990.

The Wainwright design planning process as presented by Charles H. Donaldson, operations manager, involves all key stakeholders, including suppliers and customers. The Wainwright philosophy, according to Mr. Donaldson, is, “If it ain’t broke, break it, and fix it better. The ‘fix it better process’ is based on front-end risk factor projections, an ongoing validation process matching reality against projections, and root cause analyses.” The planning approach is a collection of subsystems “that focuses on the processes and not on people.”

The risk factor evaluation, known at Wainwright Industries as Process Failure Mode and Effects Analysis, attempts to identify the causes and effects of potential failure, process capability, long term performance, and supplier capability. The validation process attempts to measure both short and long term process capability, delivery success, cost, supplier performance, design process improvements, and business support units performance.

Even the best planning projections and ongoing validation process are no guarantee of smooth sailing so Wainwright Industries instituted an eight-step root cause analysis structure to insulate the customer from disruption and surprises. Wainwright Industries termed its root cause analysis the Opportunity Resolution and Reporting Process. This eight-step, cross-functional, team-based process deals with cause and solutions to waste and non-value-added activities. This process also evaluates the effectiveness of waste elimination paths. This process can be used to measure supplier quality based on delivery time, defects, and cost. The effective implementation of these processes have produced the following important results:

- On time delivery increased 28 % in the last 3 years, within 1.8 % of the 99.3 % benchmark.
- Rejected parts measured in parts per million(ppm) have dropped 87.8 % over the last 3 years. The incident rate has been reduced from 1230 per million to 150 per million by using the Opportunity Resolution and Reporting Process and bar code reading technology.
- Sequenced product quality was within 0.055 of the 99.94 benchmark.
- Equipment utilization improved from 55 % to 75 %.
- Production lead time dropped from 8.75 days to 15 minutes.
- Freight cost, as percent of sales, was reduced 76.9 % for the last 3 years.
- The cost of loss prevention fell 85.9 %, from $88,000 in 1990 to presently $16,000.
- Debt fell from $6.3 million in 1989 to a 1995 forecast of $4 million.
- Closing monthly financial statements took 30 days in 1989 and 4 days in 1994.
- Quality cost decreased from 10 % to 1 %.

AT&T Consumer Communications Services Frank Ianna (vice president and general manager, Network Services Division) noted that, “process management teams are central to our business.” By this Mr. Ianna meant that process teams develop, manage, measure, and improve the way AT&T CCS does business with its five key customer satisfiers; call quality, customer service, reputation, billing, and price. The critical performance measures of CVA, PVA, and EVA are used to assess how well the key customer satisfiers are being managed.

The general notion that quality processes are a business cost is disputed by the AT&T CCS record for the last few years. The AT&T CCS return on quality has been a positive return and not an outlay. As Mr. Ianna noted, “Examining actual AT&T [CCS] results, the numbers are engaging. For Customer Value Added (CVA) activities, there was an 80 % to 90 % reduction in service interruption and similar types of customer problems and a decrease of 63 % in price and 90 % improvement in time to market. Looking at Economic Value Added (EVA) results, operating income has increased 10 % for the year, revenue has also increased 6 % for the same period, and cost has decreased 25 % during the same time.” Mr. Ianna continued, “In the area of AT&T CCS People Value Added (PVA), associates attitude towards training has improved dramatically in the last two years and now exceeds the high performing norm. Our people continue to participate heavily in internal suggestion and feedback programs.”

Beth Bronner, AT&T vice president, Domestic Consumer Communications Services, noted that “nothing enhances decision making more than having people working hand in hand with day to day operations on the firing line.” Process management teams meet at least monthly to assess ongoing processes. The recommendations of these teams often improve productivity, cut cycle time, and reduce waste.

## 7. Customer Focus and Satisfaction

Dr. Harry Hertz of the NIST Office of Quality Programs explained the Baldrige Criteria for customer focus and satisfaction as, “Examining the company’s systems for customer learning and for building and maintaining customer relationships. Also examined are levels and trends in the key measures of business success—customer satisfaction and retention, market share, and satisfaction relative to competitors.”

According to Michael Simms, Wainwright Industries plant manager, the customer satisfaction philosophy at Wainwright is, “Measure the ultimate success of the continuous improvement process through customer perception. The corporate goal is to be a preferred supplier for each customer.” The Wainwright motto could be “Continuous Commitment to Our Customers’ Future.”

To guarantee continuous commitment to customers, each Wainwright Industries’ customer has a customer advocate whose main responsibility is to ensure the particular customer is always heard. The customer advocate also is responsible for ensuring that the red warning flags, which Wainwright Industries uses to dramatize less than full customer satisfaction, are quickly replaced with the fully satisfied customer green flag. Each advocate has important input to processes which are addressing Wainwright’s four customer critical success factors, the best price, no rejects, on-time delivery, and partnering.

To manage the customer relationship, Wainwright measures customer satisfaction in a number of ways, gaging perceptions, frequency of complaints, producing a report card, fostering a teacher/student relationship, and monitoring its own customer satisfaction index (CSI) rating (and report card) system. Wainwright uses internal and external measures to determine both present customer satisfaction and potential customer satisfaction trends.

External indicators of customer satisfaction are: degree of success in gaining preferred supplier certifications, measuring and comparing defective parts per million (ppm) with industry trends, and monitoring carefully market share.

Internal indicators of customer satisfaction are: the customer satisfaction index (and report card) for delivery, product quality, communication, the degree of success with resolving complaints, and service—100 % (zero complaints) for all categories earns an A, 90 % is a B, and anything lower is a failing grade.

George Burnett, AT&T CCS general manager, marketing communications noted, “The customer’s voice should shape everything we do. Part of ‘Our Common Bond’ is our pledge to each customer. We truly care for each customer. We build enduring relationships by understanding and anticipating our customers’ needs and by serving them better each time than the time before. AT&T customers can count on us to consistently deliver superior products and service that help them achieve their personal and business goals.”

AT&T CCS has evolved from a pre-1990 focus on products to an early 1990s market focus to the present customer life cycle focus. Five key drivers (and marketplace success measures) of customer satisfaction, known AT&T CCS as Customer Value Added, are: reputation, price, call quality, billing, customer service.

Forty-four thousand AT&T CCS associates are data gatherers of customer satisfaction. They are taught customer contact skills to help them handle and measure the 4 million daily direct personal contacts with customers. With so many contacts, the ability to maintain strong customer relationships is critical.

Information from direct customer contacts, environmental research (which is a scan of external factors such as the industry as a whole, the competition, future customer trends, and projected technology), and from the hands-on experiences in AT&T test labs of over 10,000 customers each year decrease the degree of risk and increase the potential for customer satisfaction associated with present and future products and services. Mr. Burnett summarized the AT&T CCS approach to customer focus and satisfaction as, “Listen to the customer, link customer experiences and expectations to processes, and continuously reinvent and innovate.”

“Some years ago, as GTE Directories was starting its Baldrige journey,” Marilyn B. Carlson, GTE Directories’ senior vice president, shared with the audience, “The company had been experiencing a great deal of change, change that was largely a by-product of the Malcolm Baldrige Criteria. We had invested several years in the Baldrige process and we had made a lot of progress in adapting our business to the Baldrige Criteria. But sometimes change is difficult. This category, in particular—Customer Focus and Satisfaction—was steering us towards a profound transformation in our approach to sales and customer service. As we stood at the threshold of change, we faced a decision. Either keep doing business the traditional way, which had worked rather well for 50 years, or trust the Baldrige process and findings of our customer satisfaction research and go forward with substantial changes. As you can probably guess, we made the leap of faith. And in doing so we set off a chain of events that cascaded through our company like a line of dominoes.”

Ms. Carlson explained that the corporate vision of “100 % Customer Satisfaction Through Quality” is only a few years old. Before this the, GTE Directories vision was, “to be the number one directories publisher in the world.” As she noted, “The main problem with the old vision was that it begged the question ‘number one in what?’ Number one in revenues? Number one in volume of sales? We never really determined that number and what the priorities should be. However the more we adapted ourselves to the Baldrige Criteria, the more we realized that this was a question for our customers to decide. In other words, we should find out what our customers’ priorities are and make those priorities our own.”

GTE Directories approaches this vision by addressing a group of customer-based priorities: provide and demonstrate value, build business relationships, enhance customer service, improve cost effectiveness to enhance competitiveness. GTE Directories’ uses seven drivers of customer satisfaction to attack these priorities: product quality, documentation, ad content, sales, business relationships, customer service, and value. Ms. Carlson noted that GTE Directories uses a series of measures to determine five areas of customer information.

- What do our customers want and expect from us?
- How well are we meeting their needs compared to our competitors?
- What aspects of our products and services are most important?
- How will quality improvements increase customer satisfaction?
- Is our level of customer satisfaction improving?

Each GTE Directories sales professional is trained to apply the specific group of customer contact steps with each customer. Complementing and backstopping the customer contact process is an equally systematized customer complaint resolution process which GTE Directories sees as fundamental to its customer satisfaction efforts. The customer complaint resolution process is supported by a toll-free number and the corporate goal of “First-Call Resolution.” This process kicks in when a complaint is received. But the company does not wait until complaints surface. It surveys its customers regularly and directly asks each customer to rate the services and value being received.

Ms. Carlson concluded by observing, “Our results show that we are indeed making progress. Having made the leap of faith, our quality improvement initiatives are improving customer satisfaction, and that means we are all well on our way to achieving our 100 % customer satisfaction through quality vision.”

## 8. Lessons Learned

Diane Phelps-Feldkamp, AT&T Consumer Communications Services, director, quality and business improvement led off the session with her observations leading up to winning the Baldrige Award as well as after winning. Lessons on the way to winning the Baldrige Award:

In general, dedicate yourself to the customer.
Treat customers as individuals.
Value your people.
Constantly reinvent the organization.
Focus on the customer.
Know your customer satisfiers.
Understand customer potential
– mix of product usage
– emotional factors
– overall behaviors.

Lessons from the Baldrige experience:

The AT&T “Quality” heritage created a greater challenge.
Top management must be committed to incremental improvement.
Only the right data count.
Quality becomes the nature of the company, rather than a parallel process (“Quality” titles on the organization chart significantly reduced).
Motivate through respect and involvement.
The experience re-energizes.
Communication is key.
The Baldrige Criteria provide for the ultimate self-assessment guide.
Guidelines drive common goals.
Baldrige is contagious.
Tackle one part of the elephant at a time.

Ms. Phelps-Feldkamp summarized the AT&T CCS lessons learned as: focus on the customer, focus on the workforce, reinvent, and don’t fear competition.

According to James Runyon, GTE Directories director—quality services, their lessons on the way to the Baldrige were:

Build a quality improvement infrastructure based on the Baldrige experience (the Baldrige Criteria provided the perfect foundation guide).
Vice presidents named Baldrige “champions.”
Nineteen initial Baldrige teams were formed.
More than 800 employees participated in the eventual 106 Baldrige teams.
Seventy-five process management teams were formed.
To date, 6435 quality improvement teams were formed.
There has been 100 % employee participation in Baldrige self-assessment process.
Created a quality central team which compiled and edited reports from Baldrige teams, prepared Baldrige application for executive management review, began preparations for site visit by Baldrige examiners, and managed all site visit activity.

Mr. Runyan considered the Baldrige process as important because:

It provides a framework for quality improvement.
It is a motivator.
It breeds “healthy discontent.”
It is a catalyst for change.
It promotes teamwork.

Mr. Runyan added, “Build relationships with suppliers, holding them to the same standards of quality, benchmark against industry standards and key competitors, and don’t ever fear the competition.” Summing up the Baldrige experience for GTE Directories, Earl Goode observed, “The greatest thing about winning the Baldrige Award is that now we have to act like a Baldrige Award winner. And I can see the winning attitude in the faces of our employees every day. I can see it in the new ways they approach challenges, and I can see it in the stronger partnerships we are beginning to form with our customers.”

While AT&T CCS and GTE Directories presented lessons learned from the experience of building quality organizations, W. Nelson Wainwright II, president, Wainwright Industries, interspersed his lessons learned with an anecdotal flavor of what applying for Baldrige Award was like for his firm. The following paragraphs present excerpts from the Wainwright journey (a journey that started in 1981 when Wainwright Industries and all other prime General Motors suppliers were challenged by then GM CEO Roger Smith to produce world class quality products or be removed from the GM team).

Mr. Wainwright explained, “All of who have read or studied a Baldrige application know that it can be intimidating, and we were no different. Since 1990, whenever the subject of applying for the Baldrige came up, folks at Wainwright Industries seemed to always find reasons to not apply. Some excuses:

It interferes with production.
The application process is too complicated. (The first Wainwright Industries Baldrige application was written in a weekend.)
It is too costly to apply.
We do not have enough time and resources to devote to the process.
We have no chance or do not deserve to win.

Lesson learned: There’s no reason to be intimidated by the application or the application process. Writing an application serves as a great learning experience for any company. The knowledge gained makes a company a winner even if it doesn’t get a site visit or win the Award. I wonder how many great companies, and yours may be one, could have won the Award but never applied because they did not feel they were good enough relative to the application criteria?”

“Having applied for both the Malcolm Baldrige National Quality Award and the Missouri Quality Award in 1993, we learned a great deal about the Baldrige criteria and ourselves that allowed us to write a far more concise application in 1994. If your state has a quality award, go for it. Even if the state criteria do not exactly fit the Baldrige criteria, the application and site visit process will be very beneficial to learning about your organization and preparing for the Baldrige. Lesson learned: The right practice makes perfect.”

Other key lessons shared by Mr. Wainwright are:

“It pays to listen to associates because we all can benefit greatly from their front line, in-the-trenches knowledge. Encouraging small suggestions will unleash amazing creativity in associates that will result in tremendous cost savings and efficiency improvements. Tapping the creativity of each associate through the CIP process makes it very difficult for competitors to keep pace.
By creating an environment where associates will take risks and they know how they can contribute, results in their feeling responsible for the success of the total enterprise and true empowerment is achieved.
Benchmarking with a competitor can be a win-win situation by giving both entities new ideas and making both more competitive in the world marketplace.”

In conclusion to the lessons learned session, Ms. Phelps-Feldkamp of AT&T CCS probably articulated the general feeling of all Baldrige winners, past and present, by noting that, “success is never final.”

## 9. Quality Pays

This year’s conference included as its final plenary session a keynote address and panel discussion led by representatives from past Baldrige-winning companies. They shared experiences which could be characterized as “life after Baldrige.”

Session moderator Daniel Burton, president of the Council on Competitiveness, noted that the panelists, “… leave us with the impression that there is life after the Baldrige, in fact life is pretty good after winning the Baldrige. In general, all four companies represented on today’s panel thrived, as measured by hard core business indicators such as profit and market shares and return on equity. They also focused and thrived on the soft side of their business, for instance customer loyalty, employee job satisfaction, and return on human resource assets. And, as is true with this year’s Baldrige winners, all four companies on the panel today thrive on competition.”

Keynote speaker, Dr. Koichi Nishimura, president and CEO, Solectron Corporation, a 1991 Baldrige Award winner, spoke on customer-driven agile manufacturing, which he defined as “sustainable ability to consistently thrive and profit in an environment of unpredictable and rapid changes.” Quoting Dr. Nishimura, “You can look at product-out or market-in when trying to determine the health of your business. But you will find that the product-out approach days are over. These are the days of customer-driven, market-in orientation … what does the customer want. In the old days we split the cost and put the profit on, those days are over. Customers want things better, cheaper, faster and they want it now.

“The Japanese view the product life cycle as 3-9-1, 3 month development cycle, 9 month product life, and 1 month end of life. Only a very agile organization will be able to exist in a world with such tight product cycles and short term product lives.” Using a Solectron plant as an example of agile manufacturing, Dr. Nishimura noted that it produces 800 different printed circuit board assemblies every day, in lot sizes from five to two thousand, two thirds of which will have to be changed by the next day based on new customer requirements. Dr. Nishimura observed, “So what gets staged tomorrow for us, two-thirds will be different than what we are looking at on paper today. Linear programming does not work any more because it assumes that time is an invariant. An agile company is one which uses a dynamic form of programming recognizing time as a key variable.”

Dr. Nishimura closed with, “At Solectron, we use the Baldrige Criteria to remain an agile corporation. The Criteria created a focus for us, a shared vision company wide. The rigor of the process helped us, we learned how to benchmark and borrow from the best. The examiner feedback was great and we worked on every area needing improvement. And our profits and revenues have been growing every year.”

The panel portion of the final session was led off by Ray Marlow, president of Marlow Industries, a 1991 Baldrige winner. Mr. Marlow noted that his company is in the midst of a growth period, increasing personnel 60 % and productivity 33 % since winning the Baldrige in 1991. According to Mr. Marlow, “The biggest challenge during this period has been in maintaining a TQM culture.” Mr. Marlow also noted, “The Baldrige has also given a boost to our name recognition.”

Panel member Patrick Mene, vice president of quality, Ritz-Carlton Hotel Company, a 1992 Baldrige winner, noted that “… even after receiving the Baldrige Award, the examiners’ feedback and the Criteria have played major roles in cutting cycle time by 50 %, defects twofold, and increasing customer satisfaction by 50 %.” According to Mr. Mene, improvement in cycle time, defects, and customer satisfaction have led to increased customer loyalty, hotel expansion, significant reduction in costs, and significant increases in revenue. Mr. Mene noted that *Consumer Reports* in 1994 rated Ritz-Carlton the world’s number one hotel. “Market yield, as a measure of revenue per available room, is best in industry,’ according to Mr. Mene, “and profitability is best in class (among high grade hotels).”

Robert Osterhoff, director of corporate quality, Xerox Corporation, a 1989 winner, noted in his panel remarks that Xerox stock is at a 20 year high and return on assets has reached a 15 year high. Mr. Osterhoff emphasized, “Xerox 2000, Leadership Through Quality, supporting the Xerox Management Model with its customer and market central focus, is one of the most important steps taken since achieving the Baldrige Award. Essentially this is a Baldrige system in action.”

Randy Quint, director of market driven quality, IBM Rochester, a 1990 winner, noted that by any measure and all measures, internal and external, quality pays. Citing a 1993 *Business Week* study, Mr. Quint said, “If you are going to throw a dart at a stock board, you hope to hit Baldrige companies. This *Business Wee*k survey was just updated in February and publicly traded Baldrige companies are outpacing the Standard & Poor 500 by three to one.” Mr. Quint further observed, “The most dramatic shift in IBM since winning the Baldrige has been the present customer-view model orientation. We used to run our business and look at processes based upon how we viewed them. We made a fundamental shift asking our customers how they view us. And now we are looking at our processes through our customers’ eyes.”

## 8. Summary

In his closing remarks to the conference, Dr. Curt W. Reimann observed, “What this conference is really about is improving business performance in the United States. If there’s a story that needs repeating as part of this Award program and the engine that really keeps it going, it is not only the results of the companies that have won, but their willingness to share their time and experiences, in some cases, five or six years later. That story is beyond our wildest dreams and expectations when we were working on this process in 1987.” Dr. Reimann further noted that Baldrige Awards recipients, even going as far back as 1988, have been involved in “literally thousands of events, sharing lavishly for seven years and without government support of any kind.”

Reinforcing Dr. Reimann’s comments, Don Wainwright helped to summarize and capture the flavor of this year’s Quest for Excellence Conference by observing, “The one thing you learn through the years with the Baldrige process is that the more you share, the more you learn, and the more you actually gain. So you really become a disciple of the quality movement and you want to spread the good things you have done with other people.”

## 9. General References

1994 Criteria for the Malcolm Baldrige National Quality Award.

Conference notebook, Quest for Excellence VII, February 6–8, 1995, Washington, DC.

Audio Archives International tapes of the conference.

